# Dual role of calcium-activated potassium channels of high conductance: facilitator or limiter of NO-induced arterial relaxation?

**DOI:** 10.3389/fphys.2025.1563014

**Published:** 2025-03-27

**Authors:** Anastasia A. Shvetsova, Dina K. Gaynullina, Johannes Schmid, Peter Winkler, Isabella Sonsala, Rudolf Schubert

**Affiliations:** ^1^ Faculty of Biology, M.V. Lomonosov Moscow State University, Moscow, Russia; ^2^ Insitute of Physiology, Russian National Research Medical University, Moscow, Russia; ^3^ Physiology, Faculty of Medicine, Institute of Theoretical Medicine, University of Augsburg, Augsburg, Germany; ^4^ Research Division Cardiovascular Physiology, Medical Faculty Mannheim, European Center of Angioscience (ECAS), Heidelberg University, Mannheim, Germany

**Keywords:** vasodilation, arterial smooth muscle, BK channel, nitric oxide, tail artery, saphenous artery, coronary arteries

## Abstract

**Aim:**

Calcium-activated potassium channels of high conductance (BK_Ca_ channels) are important contributors to vascular smooth muscle membrane potential and thus to vascular tone. BK_Ca_ channels can promote vasodilation by facilitating vessel responses to NO. BK_Ca_ channels may also serve as limiters of the anticontractile effect of NO. However, it is unclear whether BK_Ca_ channels act simultaneously as facilitators and limiters in different vascular regions. Therefore, this study tested the hypothesis that BK_Ca_ channels both facilitate and limit NO-induced vasorelaxation in multiple vessels.

**Methods:**

Contractile responses of rat tail, saphenous, and left and right coronary arteries were studied using wire myography.

**Results:**

The NO-donor SNP reduced contractile responses induced by low concentrations of methoxamine or serotonin, respectively, in all arteries tested, both in the absence and in the presence of iberiotoxin. This anticontractile effect of SNP was larger in the presence of iberiotoxin than in its absence, i.e., functionally active BK_Ca_ channels limit the anticontractile effect of SNP. In contrast, the anticontractile effect of SNP at high concentrations of methoxamine or serotonin, respectively, in all arteries tested was smaller in the presence of iberiotoxin than in its absence, i.e., functionally active BK_Ca_ channels facilitate the anticontractile effect of SNP.

**Conclusion:**

BK_Ca_ channels simultaneously limit NO-induced vasodilation at lower levels of contractility but facilitate it at higher levels of contractility in multiple vascular beds. Therefore, BK_Ca_ channels may play a dual role as facilitators and as limiters of the effect of NO, depending on the level of contractility.

## 1 Introduction

The regulation of blood pressure and organ perfusion is largely determined by the diameter of the arteries in the circulatory system. Arterial diameter is substantially governed by the membrane potential of vascular smooth muscle cells. Potassium channels, in particular the calcium-activated potassium channels of high conductance (BK_Ca_ channels), make an important contribution to the membrane potential. Under physiological conditions, these channels have been observed to serve as negative feedback for vasocontraction and to contribute to vasodilation (Tykocki et al., 2017). They have also been reported to be involved in several diseases such as hypertension and diabetes (Lu et al., 2005; McGahon et al., 2007; Kyle and Braun, 2014). Thus, vascular smooth muscle BK_Ca_ channels are important contributors to physiological regulation as well as pathophysiological dysregulation in the circulatory system.

Regarding the contribution of BK_Ca_ channels to vasodilation, they have been shown to facilitate vessel responses to NO (Williams et al., 1988; Khan et al., 1993; Robertson et al., 1993) in many vascular beds; sometimes BK_Ca_ channels have been reported to be resistant to NO-induced regulation (Tykocki et al., 2017). In addition, a recent study presented data showing that BK_Ca_ channels can also serve as limiters of the anticontractile effect of NO; this has been described for rat and mouse tail and rat saphenous arteries (Schmid et al., 2018). Mechanistically, this role of the BK_Ca_ channel has been explained by the existence of two simultaneous effects of NO on BK_Ca_ channels: channel activation mediated by PKG (already established for some time by a number of studies (Alioua et al., 1995; Schubert and Nelson, 2001; Kyle et al., 2013)) and channel deactivation mediated by a decrease in the intracellular calcium concentration. It has been suggested that, particularly at lower levels of vessel contractility, NO-induced PKG-mediated activation of the BK_Ca_ channel is weaker than NO-induced [Ca^2+^]_i_ decrease–mediated deactivation of the BK_Ca_ channel and that the overall decrease in BK_Ca_ channel activity determines the role of BK_Ca_ channels as limiters of the effect of NO. Of note, this mechanistic framework could also explain the previously described role of the BK_Ca_ channel as a facilitator of the effect of NO. It was suggested that at higher levels of vessel contractility, NO-induced PKG-mediated activation of the BK_Ca_ channel is stronger than NO-induced [Ca^2+^]_i_ decrease–mediated deactivation of the BK_Ca_ channel and that the overall increase in BK_Ca_ channel activity determines the role of BK_Ca_ channels as facilitators of the effect of NO. BK_Ca_ channels may therefore play a dual role, namely, as facilitators and as limiters of the effect of NO. However, it is unclear whether a simultaneous presence of the facilitator and limiter role of the BK_Ca_ channel is a more general phenomenon in the circulatory system, i.e., can be observed in different vascular regions. Therefore, this study tested the hypothesis that BK_Ca_ channels both facilitate and limit NO-induced vasorelaxation in multiple vessels.

## 2 Materials and methods

### 2.1 Animals

All experimental procedures in this study complied with the European Convention on the protection of animals used for scientific purposes (EU Directive 2010/63/EU) and were approved by German institutional committees on animal welfare (I-17/17). Male Wistar rats were used in this study. Animals were obtained from Janvier (France), aged 2–3 months and weighted 250–350 g. Rats were housed in a room with a controlled temperature and a 12/12 h light/dark cycle with free access to water and food *ad libitum*. At the day of the experiment animals were anesthetized by CO_2_ and decapitated.

### 2.2 Wire myograph experiments

The tail, saphenous, right and left coronary arteries were utilized in this study. Arteries were carefully isolated from the surrounding tissue, each type of artery was cut into four segments and mounted in a wire myograph (620M, DMT A/S, Denmark). The endothelium was carefully removed using a rat whisker. All preparation procedures were carried out in the preparation solution containing (mmol L^−1^): NaCl 145; KCl 4.5; CaCl_2_ 0.1; MgSO_4_ 1.0; NaH_2_PO_4_ 1.2; EDTA 0.025; HEPES 5.0 (pH = 7.4).

Data from our previous study describing that BK_Ca_ channels may also serve as limiters of the anticontractile effect of NO (Schmid et al., 2018), which provide a mechanistic background for the present study, were obtained using only male rats. Because the present study is closely related to our previous study, especially in terms of mechanistic background, this study is also limited to male rats. However, we are planning such experiments for the future.

The tail artery was used because the data from our previous study describing that BK_Ca_ channels may also serve as limiters of the anticontractile effect of NO (Schmid et al., 2018) were obtained from this vessel, providing a mechanistic background for the present study. The saphenous artery was used because in several previous studies we were able to characterize the functional role of smooth muscle potassium channels, including their participation in SNP-induced vasodilation in detail (Shvetsova et al., 2019; 2025; Ma et al., 2020). The coronary arteries were used as vessels of a more “specialized” vascular bed in which NO plays an important role both under physiological (blood flow regulation) as well as pathophysiological (therapeutic use of nitrovasodilators) conditions. There are several additional vascular beds of interest (e.g., mesenteric, cerebral, renal) that should be investigated in future studies. After mounting of the vessels, the solution was replaced by experimental solution containing (mmol L^−1^): NaCl 120; NaHСO_3_ 26; KCl 4.5; CaCl_2_ 1.6; MgSO_4_ 1.0; NaH_2_PO_4_ 1.2; D-glucose 5.5; EDTA 0.025; HEPES 5.0 (pH = 7.4). The temperature in the chambers was heated up to 37.0°C and was maintained at this level throughout the experiment. To maintain pH = 7.4 the chambers were continuously aerated with a mixture of 5% CO_2_ + 95% O_2_. Data were recorded at 1 kHz using the PowerLab 4/30 system (ADInstruments, United States) and the LabChart software (ADInstruments, United States). All arterial segments were stretched to 0.9d_100_ (90% of the inner diameter it would have at a transmural pressure of 100 mmHg), corresponding to maximum active force development (Mulvany and Halpern, 1977).

**FIGURE 1:**
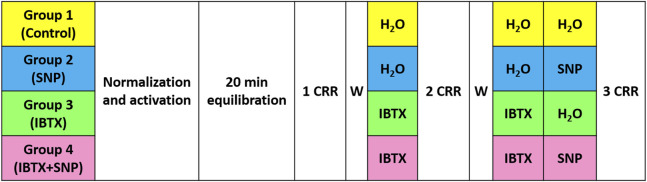
Schematic representation of the experimental protocol used in this study, see text for details. CRR–concentration response relationship, W–washout, IBTX–iberiotoxin, SNP–sodium nitroprusside.

The experimental protocol consisted of the standard activation procedure followed by three concentration-response relationships to vasoactive agonists (Figure 1). For tail and saphenous arteries the following activation procedure was performed: (1) methoxamine (α_1_-adrenoceptor agonist, 10 μmol L^−1^) for 5 min; (2) acetylcholine (10 μmol L^−1^, 2 min) on top of methoxamine-induced pre-contraction (1 μmol L^−1^, 5 min) - the absence of a dilatory response confirmed successful endothelium denudation; (3) methoxamine (10 μmol L^−1^) for 5 min. 20 min after the end of the activation procedure the first concentration-response relationship to methoxamine was obtained (concentration range from 0.01 μmol L^−1^–10 μmol L^−1^, each concentration for 3 min). This concentration-response relationship was used to ensure similar initial sensitivity to agonist in preparations further treated with a blocker and/or NO-donor. After washout of methoxamine two of four preparations were treated with the BK_Ca_ channel blocker iberiotoxin (0.1 μmol L^−1^) for 20 min, the other two preparations were treated with the same volume of water (solvent of iberiotoxin). Thereafter, the second concentration-response relationship to methoxamine was obtained in the same manner as the first one. The second concentration-response relationship was used to ensure similar responses in the groups treated with iberiotoxin and solvent, respectively. After washout of methoxamine, the preparations were treated for 20 min with either (1) iberiotoxin (0.1 μmol L^−1^); (2) sodium nitroprusside (SNP, 0.1 μmol L^−1^); (3) iberiotoxin (0.1 μmol L^−1^) + SNP (0.1 μmol L^−1^); (4) the same volume of water (solvent of iberiotoxin and SNP, labeled as Control in the graphs). Importantly, at this stage, the application of iberiotoxin was carried out only to those segments to which iberiotoxin had been added previously. Thereafter, the third concentration-response relationship to methoxamine was obtained in the same manner as previously. The third concentration-response relationships are presented in the graphs. The right and left coronary arteries were activated by application of (1) serotonin (10 μmol L-1, 5 min) followed by acetylcholine (10 μmol L^−1^, 2 min); (2) high-potassium solution containing (mmol L^−1^): NaCl 6; NaHСO_3_ 26; KCl 118.5; CaCl_2_ 1.6; MgSO_4_ 1.0; NaH_2_PO_4_ 1.2; D-glucose 5.5; EDTA 0.025; HEPES 5.0 (pH = 7.4) for 5 min; (3) serotonin (10 μmol L^−1^, 5 min). Thereafter, a similar to tail and saphenous arteries protocol was applied except serotonin was used instead of methoxamine.

Vessel reactivity was expressed as active force. To calculate active force values at each time point of interest, the force value of the fully relaxed state was subtracted from all recorded data. Further, all active force values were expressed as the percentage of the active force developed during the last step of the activation procedure (i.e., the response to 10 μmol L^−1^ of methoxamine for tail and saphenous arteries or to 10 μmol L^−1^ of serotonin for right and left coronary arteries, Table 1). Areas under the curve (AUC) values were calculated for the third concentration-response relationships in GraphPad Prizm 9.5.1 (La Jolla, CA, United States). To obtain the values of the anticontractile effect of SNP in the absence and in the presence of iberiotoxin, the AUC values in the presence of SNP alone or together with iberiotoxin were subtracted from control or iberiotoxin groups, respectively.

**TABLE 1:** Vessel tension obtained during the last step of the activation procedure in response to 10 μmol L^−1^ of methoxamine or serotonin.

Tension (mN/mm)				
Experimental group	Tail artery	Saphenous artery	Left coronary artery	Right coronary artery
Control	36.3 ± 4.6	32.6 ± 3.9	2.8 ± 0.6	3.8 (2.9; 6.2)
IBTX	43.3 ± 3.4	36.6 ± 2.4	4.3 ± 0.7	4.0 (3.3; 8.3)
SNP	42.2 ± 3.6	35.9 ± 3.1	3.1 ± 0.3	4.5 (3.0; 5.5)
IBTX + SNP	40.9 ± 3.6	37.4 ± 3.8	3.9 ± 0.3	4.1 (3.2; 5.3)
Statistical analysis	n = 10; p = 0.59 one-way ANOVA	n = 8; p = 0.75 one-way ANOVA	n = 10; p = 0.14 one-way ANOVA	n = 9; p = 0.95 Kruskal–Wallis

### 2.3 Drugs

Methoxamine, serotonin, acetylcholine, SNP (all dissolved in H_2_O), as well as all salts were obtained from Sigma. Iberiotoxin (dissolved in H_2_O) was obtained from Alomone Labs.

### 2.4 Statistical analysis

Statistical analysis was performed using GraphPad Prism 9.5.1. The normality of the data distribution was tested using the Shapiro-Wilk test. Data are presented as mean and SEM (if data distribution was normal) or as median and interquartile range (if data distribution was different from normal); *n* represents the number of animals, i.e., biological replicates (only one vessel from one animal was used in each group). Concentration-response relationships between groups were compared using repeated measures ANOVA followed by two-stage linear step-up procedure of Benjamini, Krieger and Yekutieli controlling for false discovery rate. Statistical analyses of SNP’s anticontractile effect in the absence and in the presence of iberiotoxin was carried out using unpaired t-test with Welch’s correction or Mann-Whitney U-test, depending on the type of data distribution. Differences were accepted as statistically significant if the P-value was less than 0.05.

## 3 Results

### 3.1 Tail artery

**FIGURE 2:**
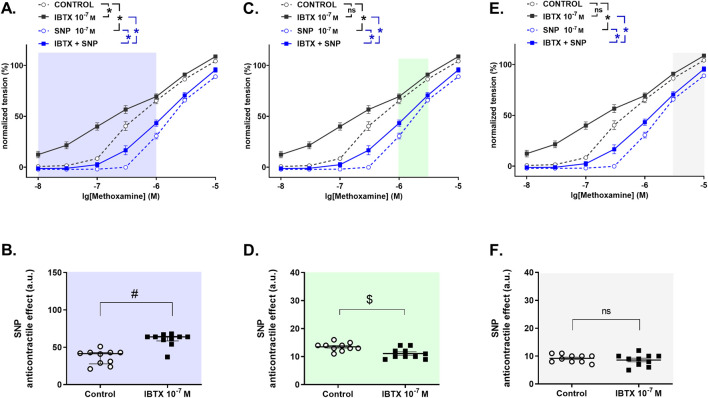
Anticontractile effect of SNP in the absence and presence of BK_Ca_ channel blockade at different levels of contractility in rat tail arteries. **(A, C, E)** Concentration–response relationships for methoxamine in the absence of any additional substances (control), in the presence of iberiotoxin (0.1 µM, IBTX), sodium nitroprusside (0.1 µM, SNP) or iberiotoxin together with sodium nitroprusside (IBTX + SNP). Data analysis was limited to the respective highlighted areas corresponding to low **(A)**, middle **(C)** and high **(E)** levels of contractility. n = 10; * - p < 0.05 (repeated measures ANOVA with two-stage linear step-up procedure of Benjamini, Krieger and Yekutieli). **(B, D, F)** Anticontractile effect of SNP in the absence (circles, Control) and in the presence of IBTX (squares, IBTX), presented as the difference between the area under the concentration-response relationships in the control and the SNP groups as well as in the IBTX and the IBTX + SNP groups. n = 10; # - p < 0.05 (Mann-Whitney test), $ - p < 0.05 (Welch’s t-test), n.s. – non-significant.

To determine the role of BK_Ca_ channels in the anticontractile effect of NO, a detailed analysis was performed to search for those levels of contractility where BK_Ca_ channels either facilitate or limit the effect of NO. The so found levels of contractility are marked with different colors in Figure 2. In the methoxamine-concentration range between 0.01 µM and 1 μM, treatment of rat tail arteries with the BK_Ca_ channel blocker iberiotoxin increased contractile responses to methoxamine (Figure 2A). The NO-donor SNP reduced methoxamine-induced contractile responses both in the absence and in the presence of iberiotoxin (Figure 2A). The anticontractile effect of SNP was larger in the presence of iberiotoxin than in its absence (Figure 2B), i.e., functionally active BK_Ca_ channels limit the anticontractile effect of SNP. In contrast, in the methoxamine-concentration range between 1 µM and 3 μM, we did not detect an effect of the BK_Ca_ channel blocker iberiotoxin on contractile responses to methoxamine (Figure 2C). The NO-donor SNP reduced methoxamine-induced contractile responses both in the absence and in the presence of iberiotoxin (Figure 2C). The anticontractile effect of SNP was smaller in the presence of iberiotoxin than in its absence (Figure 2D), i.e., functionally active BK_Ca_ channels facilitate the anticontractile effect of SNP. Finally, in the methoxamine-concentration range between 3 µM and 10 μM, we did not detect an effect of the BK_Ca_ channel blocker iberiotoxin on contractile responses to methoxamine (Figure 2E). The NO-donor SNP reduced methoxamine-induced contractile responses both in the absence and in the presence of iberiotoxin (Figure 2F). We did not detect a difference of the anticontractile effect of SNP in the presence and absence of iberiotoxin (Figure 2F), i.e., functionally active BK_Ca_ channels do not contribute to the anticontractile effect of SNP.

### 3.2 Saphenous artery

**FIGURE 3:**
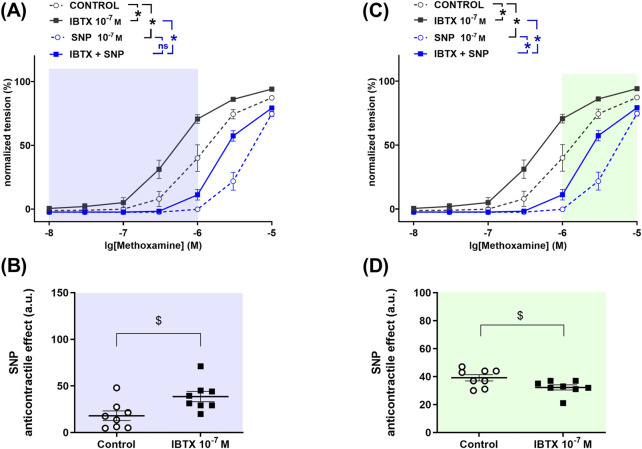
Anticontractile effect of SNP in the absence and presence of BK_Ca_ channel blockade at different levels of contractility in rat saphenous arteries. **(A, C)** Concentration–response relationships for methoxamine in the absence of any additional substances (control), in the presence of iberiotoxin (0.1 µM, IBTX), sodium nitroprusside (0.1 µM, SNP) or iberiotoxin together with sodium nitroprusside (IBTX + SNP). Data analysis was limited to the respective highlighted areas corresponding to low **(A)** and high **(C)** levels of contractility. n = 8; * - p < 0.05 (repeated measures ANOVA with two-stage linear step-up procedure of Benjamini, Krieger and Yekutieli). **(B, D)** Anticontractile effect of SNP in the absence (circles, Control) and in the presence of IBTX (squares, IBTX), presented as the difference between the area under the concentration-response relationships in the control and the SNP groups as well as in the IBTX and the IBTX + SNP groups. n = 8; $ - p < 0.05 (Welch’s t-test).

To determine the role of BK_Ca_ channels in the anticontractile effect of NO in saphenous arteries, a detailed analysis was performed to search for those levels of contractility where BK_Ca_ channels either facilitate or limit the effect of NO. The levels of contractility found in this way are marked with different colors in Figure 3. In the methoxamine-concentration range between 0.01 µM and 1 µM treatment of rat saphenous arteries with the BK_Ca_ channel blocker iberiotoxin increased contractile responses to methoxamine (Figure 3A). The NO-donor SNP reduced methoxamine-induced contractile responses both in the absence and in the presence of iberiotoxin (Figure 3A). The anticontractile effect of SNP was larger in the presence of iberiotoxin than in its absence (Figure 3B), i.e., functionally active BK_Ca_ channels limit the anticontractile effect of SNP. In the methoxamine-concentration range between 1 µM and 10 μM, the BK_Ca_ channel blocker iberiotoxin also increased contractile responses to methoxamine (Figure 3C). The NO-donor SNP reduced methoxamine-induced contractile responses both in the absence and in the presence of iberiotoxin (Figure 3C). The anticontractile effect of SNP was smaller in the presence of iberiotoxin than in its absence (Figure 3D), i.e., functionally active BK_Ca_ channels facilitate the anticontractile effect of SNP.

### 3.3 Left coronary artery

**FIGURE 4:**
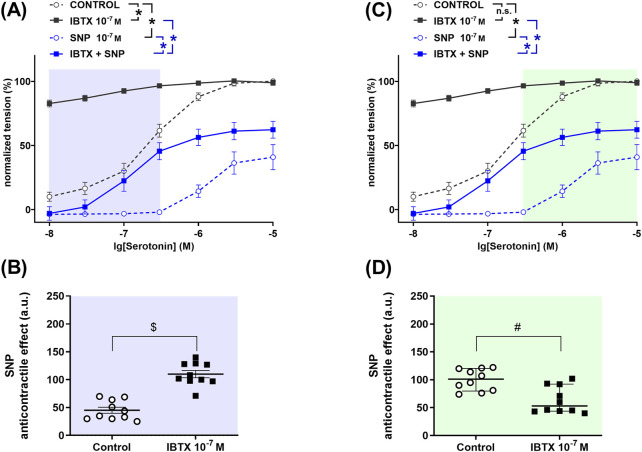
Anticontractile effect of SNP in the absence and presence of BK_Ca_ channel blockade at different levels of contractility in rat left coronary arteries. **(A, C)** Concentration–response relationships for serotonin in the absence of any additional substances (control), in the presence of iberiotoxin (0.1 µM, IBTX), sodium nitroprusside (0.1 µM, SNP) or iberiotoxin together with sodium nitroprusside (IBTX + SNP). Data analysis was limited to the respective highlighted areas corresponding to low **(A)** and high **(C)** levels of contractility. n = 10; * - p < 0.05 (repeated measures ANOVA with two-stage linear step-up procedure of Benjamini, Krieger and Yekutieli). **(B, D)** Anticontractile effect of SNP in the absence (circles, Control) and in the presence of IBTX (squares, IBTX), presented as the difference between the area under the concentration-response relationships curves in the control and the SNP groups as well as in the IBTX and the IBTX + SNP groups. n = 10; $ - p < 0.05 (Welch’s t-test), # - p < 0.05 (Mann-Whitney test).

To determine the role of BK_Ca_ channels in the anticontractile effect of NO in the left coronary artery, a detailed analysis was performed to search for those levels of contractility where BK_Ca_ channels either facilitate or limit the effect of NO. The so found levels of contractility are marked with different colors in Figure 4. In the serotonin-concentration range between 0.01 µM and 0.3 µM treatment of rat left coronary arteries with the BK_Ca_ channel blocker iberiotoxin increased contractile responses to serotonin (Figure 4A). The NO-donor SNP reduced serotonin-induced contractile responses both in the absence and in the presence of iberiotoxin (Figure 4A). The anticontractile effect of SNP was larger in the presence of iberiotoxin than in its absence (Figure 4B), i.e., functionally active BK_Ca_ channels limit the anticontractile effect of SNP. In contrast, in the serotonin-concentration range between 0.3 µM and 10 μM, we did not detect an effect of the BK_Ca_ channel blocker iberiotoxin on contractile responses to serotonin (Figure 4C). The NO-donor SNP reduced serotonin-induced contractile responses both in the absence and in the presence of iberiotoxin (Figure 4C). The anticontractile effect of SNP was smaller in the presence of iberiotoxin than in its absence (Figure 4D), i.e., functionally active BK_Ca_ channels facilitate the anticontractile effect of SNP.

### 3.4 Right coronary artery

**FIGURE 5:**
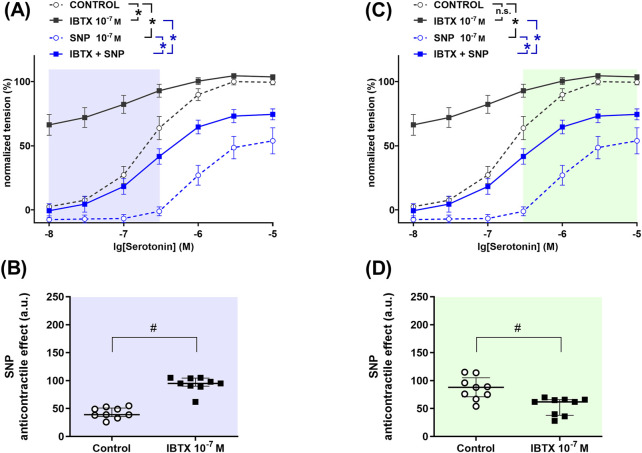
Anticontractile effect of SNP in the absence and presence of BK_Ca_ channel blockade at different levels of contractility in rat right coronary arteries. **(A, C)** Concentration–response relationships for serotonin in the absence of any additional substances (control), in the presence of iberiotoxin (0.1 µM, IBTX), sodium nitroprusside (0.1 µM, SNP) or iberiotoxin together with sodium nitroprusside (IBTX + SNP). Data analysis was limited to the respective highlighted areas corresponding to low **(A)** and high **(C)** levels of contractility. n = 9; *- p < 0.05 (repeated measures ANOVA with two-stage linear step-up procedure of Benjamini, Krieger and Yekutieli). **(B, D)** Anticontractile effect of SNP in the absence (circles, Control) and in the presence of IBTX (squares, IBTX), presented as the difference between the area under the concentration-response relationships in the control and the SNP groups as well as in the IBTX and the IBTX + SNP groups. n = 9; # - p < 0.05 (Mann-Whitney test).

To determine the role of BK_Ca_ channels in the anticontractile effect of NO in the right coronary artery, a detailed analysis was performed to search for those levels of contractility where BK_Ca_ channels either facilitate or limit the effect of NO. The so found levels of contractility are marked with different colors in Figure 5. In the serotonin-concentration range between 0.01 µM and 0.3 µM treatment of rat right coronary arteries with the BK_Ca_ channel blocker iberiotoxin increased contractile responses to serotonin (Figure 5A). The NO-donor SNP reduced serotonin-induced contractile responses both in the absence and in the presence of iberiotoxin (Figure 5A). The anticontractile effect of SNP was larger in the presence of iberiotoxin than in its absence (Figure 5B), i.e., functionally active BK_Ca_ channels limit the anticontractile effect of SNP. In contrast, in the serotonin-concentration range between 0.3 µM and 10 μM, we did not detect an effect of the BK_Ca_ channel blocker iberiotoxin on contractile responses to serotonin (Figure 5C). The NO-donor SNP reduced serotonin-induced contractile responses both in the absence and in the presence of iberiotoxin (Figure 5C). The anticontractile effect of SNP was smaller in the presence of iberiotoxin than in its absence (Figure 5D), i.e., functionally active BK_Ca_ channels facilitate the anticontractile effect of SNP.

## 4 Discussion

This study analyzed the differential contribution of BK_Ca_ channels to the anticontractile effect of NO at various levels of contractility in detail. The data obtained show that in rat tail arteries at low levels of contractility, the anticontractile effect of SNP was larger in the presence of iberiotoxin than in its absence; that at higher levels of contractility, the anticontractile effect of SNP was smaller in the presence of iberiotoxin than in its absence; and that at high levels of contractility, no difference in the anticontractile effect of SNP was detected in the presence and absence of iberiotoxin. These findings were reproduced in three other vessels, the saphenous artery and the left and right coronary arteries, where at low levels of contractility the anticontractile effect of SNP was larger in the presence of iberiotoxin than in its absence, and at higher levels of contractility, the anticontractile effect of SNP was smaller in the presence of iberiotoxin than in its absence.

Of note, NO-donors such as SNP are widely used in experimental studies and in clinical practice (Napoli and Ignarro, 2003; Miller and Megson, 2007). In the present study, SNP was used to evoke an anticontractile effect. It has been shown that the effect of SNP is very similar to the effects of NO released from the endothelium (Wanstall et al., 2001). SNP can induce effects that are independent of NO (Napoli and Ignarro, 2003). However, the NO scavenger hydroxocobalamin (Kruszyna et al., 1998), abolished the SNP-induced increase in BK_Ca_ currents (Gagov et al., 2022) and SNP-evoked vasorelaxation on rat tail arteries (Schubert et al., 2004; Schmid et al., 2018). SNP can also release cyanide (Bates et al., 1991) and/or generate nitroxyl (HNO) (Filipovic et al., 2013). However, neither substance was involved in the effect of SNP (Schubert et al., 2004; Schmid et al., 2018). The anticontractile effect of SNP is therefore most likely mediated by NO released from SNP.

In the present study, the NO-donor SNP reduced methoxamine-induced contractile responses of several arteries, which is referred as the anticontractile effect of SNP. The anticontractile effect of SNP is consistent with the well-established effect of NO as a vasodilator derived from endothelial cells (Zhao et al., 2015). BK_Ca_ channels have been shown to facilitate NO-induced vasodilation in many vascular beds (Williams et al., 1988; Khan et al., 1993; Robertson et al., 1993), and have sometimes been reported to be resistant to NO-induced regulation (Tykocki et al., 2017) (additional studies listed and discussed in (Tanaka et al., 2004; Tykocki et al., 2017)). Recently, it has been proposed that BK_Ca_ channels limit the anticontractile effect of NO (Schmid et al., 2018). The present study shows that the two roles of BK_Ca_ channels, as facilitators and as limiters of the effect of NO, co-exist, at least in the four arteries studied.

Thus, a common observation in the four vessels studied was that the anticontractile effect of SNP was larger in the presence of iberiotoxin than in its absence at lower levels of contractility, i.e., functionally active BK_Ca_ channels limit the anticontractile effect of SNP. This finding reproduces a recent report in which the role of the BK_Ca_ channel as a limiter of the effect of NO was described for the first time (Schmid et al., 2018). However, that study investigated the overall effect of NO on the full range of contractility. The data of the present study extend the previous findings by showing that the limiting role of the BK_Ca_ channel in NO-induced vasodilation is confined to lower levels of contractility. Furthermore, the present study suggests that this limiting role of the BK_Ca_ channel in NO-induced vasodilation is a more generalized property of the BK_Ca_ channel, as it was observed in four different vessels. This suggestion is supported by the observation that this role of the BK_Ca_ channel was observed in tail arteries from different species, the rat and the mouse (Schmid et al., 2018).

In a previous study (Schmid et al., 2018), data were presented showing that the limiting role of the BK_Ca_ channel in NO-induced vasodilation is mediated by the GC/PKG pathway and by an NO-induced reduction of calcium influx via L-type calcium channels. Thus, the limiting role of the BK_Ca_ channel in NO-induced vasodilation has a specific mechanistic explanation: NO exerts two simultaneous effects on BK_Ca_ channels: a PKG-mediated activation of BK_Ca_ channels, which has long been established by a number of studies (Alioua et al., 1995; Schubert and Nelson, 2001; Kyle et al., 2013), and a deactivation of BK_Ca_ channels mediated by a decrease in the intracellular calcium concentration. It was suggested that, at lower levels of contractility, the PKG-mediated activation of BK_Ca_ channels is weaker than the [Ca^2+^]_i_ decrease–mediated deactivation of BK_Ca_ channels. This leads to an overall decrease in BK_Ca_ channel activity, which defines the role of BK_Ca_ channels as limiters of the effect of NO.

Alternatively, the observed shifts in methoxamine-induced concentration response relationships could be explained by an NO-induced increase in BK_Ca_ channel activity that overcomes the iberiotoxin-induced decrease in BK_Ca_ channel activity. However, this explanation seems unlikely. Recently we reported (Ma et al., 2020) that iberiotoxin was able to completely block the shift of the methoxamine-induced concentration response relationship induced by the BK_Ca_ channel opener NS19504. Thus, although NS19504, like NO, increases BK_Ca_ channel activity, this increase in channel activity was not able to overcome the iberiotoxin-induced decrease in BK_Ca_ channel activity. More importantly, we have recently published data on BK_Ca_ currents (Gagov et al., 2022), showing that SNP does not change the BK_Ca_ current in the presence of iberiotoxin, although SNP alone produces a more than sixfold increase in BK_Ca_ channel current. Thus, although SNP alone considerably increases BK_Ca_ channel activity, this increase in channel activity was not able to overcome the iberiotoxin-induced decrease in BK_Ca_ channel activity. In conclusion, our data suggest that the observed effects represent a physiological mechanism.

Another common observation in the four vessels studied was that the anticontractile effect of SNP was smaller in the presence of iberiotoxin than in its absence at higher levels of contractility, i.e., functionally active BK_Ca_ channels facilitate the anticontractile effect of SNP. This finding is consistent with a large number of studies showing that BK_Ca_ channels facilitate NO-induced vasodilation in many vascular beds (Williams et al., 1988; Khan et al., 1993; Robertson et al., 1993), although they are sometimes reported to be resistant to NO-induced regulation (Tykocki et al., 2017), (additional studies listed and discussed in (Tanaka et al., 2004; Tykocki et al., 2017)). The data of the present study extend previous findings by showing that the facilitating role of the BK_Ca_ channels in NO-induced vasodilation is confined to higher levels of contractility. Indeed, most previous studies have investigated the effect of NO and the role of BK_Ca_ channels herein at higher levels of contractility. This was due to the fact that the experimental protocols used required pre-constriction of the arteries before the effect of the vasodilator NO could be tested. Usually, this pre-constriction was chosen to achieve higher levels of contractility as this provides more stable conditions. However, this was most likely the reason why the limiting role of the BK_Ca_ channel in NO-induced vasodilation observed at lower levels of contractility was not previously described. Thus, opposing conclusions reported in this and in previous studies do not indicate a contradiction, but are due to the fact that a common mechanism was studied under different conditions of pre-constriction.

Of note, the mechanistic framework that explains the role of the BK_Ca_ channel as a limiter of the effect of NO also explains the role of the BK_Ca_ channel as a facilitator of the effect of NO. Again, this role is mediated by the GC/PKG pathway and by an NO-induced reduction of calcium influx via L-type calcium channels, with NO exerting two simultaneous effects on BK_Ca_ channels: a PKG-mediated activation of BK_Ca_ channels, long established by a number of studies (Alioua et al., 1995; Schubert and Nelson, 2001; Kyle et al., 2013), and a deactivation of BK_Ca_ channels mediated by a decrease in the intracellular calcium concentration. It has been suggested that, at higher levels of contractility, the PKG-mediated activation of BK_Ca_ channels is stronger than the [Ca^2+^]_i_ decrease–mediated deactivation of BK_Ca_ channels. This leads to an overall increase in BK_Ca_ channel activity, which defines the role of BK_Ca_ channels as facilitators of the effect of NO.

Regarding the activity of BK_Ca_ channels, RyR-mediated opening of BK_Ca_ channels by calcium sparks has been described in cerebral and coronary artery smooth muscle cells (Nelson et al., 1995; Bychkov et al., 1997; Knot et al., 1998). However, BK_Ca_ channel activity has been shown to be independent of calcium sparks in other vessels (Szado et al., 2001; Westcott and Jackson, 2011), including the tail artery examined in the present study (Iozzi et al., 2013). Thus, the data of the present study show that the dual role of the BK_Ca_ channel in NO-induced relaxation is observed both in the presence as well as in the absence of calcium spark coupling to BK_Ca_ channels. Thus, the question of how activator calcium, supplied by calcium sparks or alternative pathways like voltage-gated calcium channels, and NO-induced signaling converge at the BK_Ca_ channels is a rather complex issue that requires a detailed and comprehensive investigation in the future.

Our previous study (Schmid et al., 2018) suggests a mechanistic framework for both the limiting and the facilitating role of BK_Ca_ channels in NO-induced vasodilation. Strictly speaking, the mechanistic explanations are limited to the tail artery, the vessel from which most of the data were obtained in the previous study (Schmid et al., 2018). Although we consider it likely that similar mechanisms are operating in the other vessels tested - at least we currently have no evidence that this is not the case - the question of whether the proposed mechanisms are common for different vessels has to be addressed. This should be done in future studies.

With our data, we were able to demonstrate a facilitator and a limiter role of the BK_Ca_ channel under conditions of vasoconstrictor-induced tone and confirm the hypothesis that BK_Ca_ channels both facilitate and limit NO-induced vasorelaxation in multiple vessels. Whether, and if so to what extent, this dual role can also be observed under other conditions (pressure-induced tone, interaction with active endothelium etc.) and ultimately *in vivo* requires further investigation.

## 5 Conclusion

In conclusion, the present study shows that BK_Ca_ channels can play a dual role, namely, as facilitators and as limiters of the effect of NO. Remarkably, both roles coexist in the same artery, albeit at different levels of contractility. The BK_Ca_ channels limit NO-induced vasodilation at lower levels of contractility, but facilitate NO-induced vasodilation at higher levels of contractility. The simultaneous presence of the dual role of BK_Ca_ channels in the same artery seems to be a general phenomenon, as it has been observed in different species, the rat and the mouse (Schmid et al., 2018) and in several different arteries (this study).

## Data Availability

The raw data supporting the conclusions of this article will be made available by the authors, without undue reservation.
